# Research on Plant Genomics and Breeding

**DOI:** 10.3390/ijms242015298

**Published:** 2023-10-18

**Authors:** Jie Huang, Zhiyong Li, Jian Zhang

**Affiliations:** State Key Laboratory of Rice Biology and Breeding, China National Rice Research Institute, Hangzhou 311400, China; huangjie@caas.cn

## 1. Introduction

In recent years, plant genomics has made significant progress following the development of biotechnology. Based on traditional genetics, functional genomics covers research on the genome, transcriptome, proteome, metabolome, bioinformatics, and other interdisciplinary disciplines. Researching many genes in the genome and their expression reveals the gene function, subcellular localization, and interaction, which provides a more systematic and comprehensive study means for the in-depth understanding of plants [1,2]. At the same time, crop breeding is crucial for agricultural development. Many high-yield and environmental adaptability crop varieties have been bred using plant genomics and gene diversity [3]. Here, we summarized some genomics studies of different species recently published in *IJMS* in the hopes of contributing to the advancement of crop breeding.

## 2. Genomics Studies in Major Food Crops

### 2.1. In Rice

Rice is one of the most important food crops in the world. Investigating the genes controlling rice yield, quality, and stress resistance using genomics leads to significant improvement. The grain length of rice seed is a crucial trait determining rice yield. In contrast, japonica rice in the Heilongjiang region of China is significantly shorter than the indica rice varieties in southern China. Two japonica rice varieties, Pin20 and Songjing 15, with significant differences in grain shape, were used to map a new QTL *qGL9.1* controlling the grain length, which promotes the study of the molecular mechanism controlling the grain length of japonica rice and lays the theoretical foundation for japonica rice improvement in northern China [4]. The salt tolerance of rice is important to improve the adaptation and yield of rice in saline–alkali soil. Low phosphate root 5 (OsLPR5) and ferroxidase activity levels were expressed during salt stress. Then, the physiological role of *OsLPR5* in salt stress was demonstrated via overexpression and using CRISPR/Cas9-mediated mutation techniques. This study identified a novel molecular mechanism in which *OsLPR5* positively regulates salt tolerance in rice [5]. Perennial rice plays a vital role in protecting the ecological environment and alleviating the labor shortage of farmers. The development of axillary buds is a crucial trait of perennial rice (regenerative rice). It was reported that sucrose promotes the growth of axillary buds and rhizomes, which might be related to fructose and glucose. The increased osmotic pressure of the cells results in the absorption of water, which encourages cell growth and eventually leads to the development of axillary buds and rhizomes [6]. The domestication of wild rice is caused by genomic variation, including the synonymy codon usage bias (SCUB) due to synonymy nucleotide substitution. SCUB was affected in the same way during the process of domestication of Asian and African rice. The analysis of cytosine to thymine transformation mediated via SCUB and DNA methylation showed the close relationship between genetic variation and epigenetic variation in domestication [7]. *LEAF TIP RUMPLED 1* (*LTR1*) positively regulates the rice yield and salt tolerance. This study showed that *LTR1* maintained the salt tolerance of rice by mediating the activities of aquaporins and ion transporters. The research on *LTR1* provides a theoretical basis for studying salt tolerance and high rice yields [8]. NF-YCs family transcription factors, including seed development, play a key role in plant growth and development. *NF-YC8*, *9*, *10*, *11*, and *12* are homologous genes that are expressed at highly similarity levels in seeds. Systematic genetic experiments suggested that these five genes synergistically regulate GA and ABA responses, thus affecting the rice seed quality and germination [9].

### 2.2. In Wheat

Wheat is the world’s largest cultivated and most widely distributed food crop. Unlike rice, wheat is a dryland crop. Therefore, it is important to improve wheat’s drought tolerance. Two previously reported KASP markers, TaDreb-B1 and 1-FEH w3, were employed for the marker-assisted selection (MAS) of drought tolerance. These two molecular markers were used to detect drought tolerance in winter and spring wheat populations, and the correlation of TaDreb-B1 with drought tolerance was better than that of marker 1-FEH w3 [10]. Cytokinin is an important hormone that determines the wheat yield. Members of the *TaCKX* gene family (GFMs) encode the cytokinin oxygenase/dehydrogenase enzyme (CKX), which causes the irreversible degradation of cytokinin. Thus, these genes might regulate wheat yield. It has been speculated that it is feasible to select high-yielding wheat families using the *TaCKX* GFMs cross-generation expression pattern [11].

### 2.3. In Maize

Maize is the world’s most productive food crop per unit area, reaching up to 30,000 kg per hectare. While abiotic stresses such as drought and salinization severely restrict maize yield, it is important to resolve the mechanism of maize flowering time and abiotic stress to improve maize yield and resistance. It has been reported that protein arginine methyltransferase (PRMTs) is mainly responsible for the histone methylation of specific plant arginine residues. Ling et al. found that the overexpression of the maize *ZmPRMT* gene in *Arabidopsis* can promote early flowering and heat tolerance. First, it was reported that *ZmPRMT1* gene regulates the flowering time and resistance to heat stress in plants, which will provide an essential theoretical basis to further reveal the functions of *ZmPRMT* gene and epigenetic regulation mechanism of maize growth and development and response to abiotic stress [12]. Corn stalks can be used as an animal feed, and sugar in the stalk is also a carbon source for corn seeds. Increasing the sugar content of corn stalks can not only promote the development of animal husbandry, but also increase the yield. Chen et al. investigated 188 waxy, sweet, and hybrid corn resources with different stalk sugar contents for GWAS analysis. The results showed that the expression levels of six candidate genes were significantly different among the other stalk sugar-containing materials, which provides a significant insight into the genomic footprints of stalk sugar content in fresh corn and facilitates the breeding of corn cultivars with a higher stalk sugar content [13].

## 3. Genome Research in Cash Crops

The production of cash crops (like cotton, soybean, rapeseed, *Brassica oleracea,* etc.) plays a decisive role in industry development, especially the light industry, which is also the main source of exports, foreign exchange earnings, and increasing national economic income.

### 3.1. In Oil Crops

Soybean sterile line SXCMS5A is an H3A cytoplasmic male sterile line (CMS) developed from JY20. Bai et al. conducted the transcriptome analysis of the sterile line SXCMS5A and the maintainer line SXCMS5B to find the differentially expressed genes (DEGs) and the metabolic pathways related to pollen sterilization. These findings might provide useful information to facilitate soybean hybrid breeding [14]. Excellent root development is an important factor for plants’ high nitrogen-use efficiency (NUE). Ahmad et al. revealed 16 genes involved in rapeseed root development under low nitrogen (LN) stress through the integrated analysis of GWAS, a weighted gene co-expression network, and DEGs. Seven of these genes have been previously reported to be associated with root development and NUE. This study provides genetic/SNPs resources for studying low nitrogen tolerance in rapeseed [15].

### 3.2. In Horticultural Crops

Khusnutdinov et al. found that the DNA binding structure of transcription factor *MYBL2-1* (a negative regulator of anthocyanin synthase *Dihydroflavonol-4-reductase* (*DFR*)) of purple varieties of *Brassica oleracea* L. had two SNP mutations, leading to an increase in *DFRs* expression. This is the reason for the high anthocyanin content of purple cabbage [16]. *Ipomoea aquatica* is a leafy vegetable rich in essential amino acids, flavonoids, and various mineral elements (calcium, potassium, phosphorus, etc.). The role of *IabHLHs* family transcription factors in the biosynthesis of anthocyanins in *I. aquatica* was analyzed using bioinformatics combined with molecular experiments. This work provides valuable clues to further explore *IabHLH*’s function and facilitating the breeding of anthocyanin-rich varieties of *I. aquatica* [17]. Improving the cold tolerance of herbaceous peonies (*Paeonia lactiflora*) is essential to plant them at low latitudes. Wang et al. modified the previously reported multi-criteria decision-making (MCDM) model. This model was implemented to analyze 15 peony varieties at different latitudes. As a result, ‘Meiju’, ‘Hang Baishao’, ‘Hongpan Tuojin’, and ‘Bo Baishao’ were excellent varieties with strong environmental adaptability. This research can provide a reference for the breeding and cultivating of perennial herbs [18]. NAC transcription factors are crucial in plant growth, development, and stress responses. Zhao et al. found that *AeNAC83* positively regulates the salt tolerance of okra (*Abelmoschus esculentus*). Exactly as the expression of *AeNAC83* gene is up-regulated in okra under salt stress, the down-regulation of *AeNAC83* is caused by virus-induced gene silencing enhanced plant sensitivity to salt stress [19]. Lotus (*Nelumbo nucifera*), which is in the Nelumbonaceae family, is a relict plant with crucial scientific research and economic values. Such as the sequencing of the lotus genome and the assembly of several high-quality genomes, the investigation of lotus functional genome has been extensively promoted. The resequencing of natural and genetic populations and different levels of genomic studies benefit the classification of different lotus germplasm resources and the identification of genes controlling various traits [20].

### 3.3. In Other Crops

Liu et al. analyzed the genetic basis of senescence in cotton using 355 upland cotton accessions planted in multiple environments for GWAS. From the candidate genes that have been linked, *GhMKK9* silencing improves the drought resistance of cotton, whereas *GhMKK9* overexpression accelerates senescence in *Arabidopsis* [21]. *Areca catechu* is a commercially important medicinal plant widely cultivated in tropical regions. Zhou et al. investigated 12 *natural resistance-associated macrophage protein* (*NRAMP*) genes from the whole genome of *A. catechu* with Fe and Zn deficiency, and their sequence characteristics, gene structure, phylogeny, promoter sequence, and collinearity were analyzed. This research revealed the regulatory response of *NRAMP* family genes in *A. catechu* under Fe and Zn deficiency stress [22]. Yao et al. treated tartary buckwheat (*Fagopyrum tataricum* L.) leaves with different concentrations of H_2_O_2_, and then analyzed the growth, photosynthesis, antioxidant enzyme activity, and related gene expression under salt stress. This study explored the mechanism of H_2_O_2_ enhancing salt tolerance of Tartary buckwheat from a physiological point of view [23].

## 4. Conclusions and Perspectives

In summary, we reviewed 20 plant genomics articles on different plant species. These functional gene studies consider the yield, quality, fertility, abiotic stress, and evolution of rice, wheat, maize, and cash crops. These researchers have made outstanding contributions to plant genomics and provided valuable genetic resources for crop breeding. Based on these genetic resources controlling different traits, gene-editing methods, such as CRISPR-cas9 technology, can alter gene expression to improve the crop yield, quality, and resistance. Finally, strengthening the interface between the application and industrialization of functional genomics research would help to accurately quantify traits, such as the crop yield, stress resistance, quality, and nutrient content. Meanwhile, promoting more efficient and precise crop breeding is expected to bring about new transformations in the global agricultural field.
